# User acceptance of telerehabilitation in Germany: a structural equation modeling approach based on the UTAUT2 model

**DOI:** 10.3389/fdgth.2026.1699317

**Published:** 2026-07-01

**Authors:** Susanne Stampa, Oliver Razum, Christoph Dockweiler

**Affiliations:** 1 Department of Social Sciences, Chair of Digital Public Health, Faculty of Arts and Humanities, University of Siegen, Siegen, Germany; 2 Department of Epidemiology and International Public Health, School of Public Health, Bielefeld University, Bielefeld, Germany

**Keywords:** acceptance, affinity for technology, structural equation modeling, telehealth, telerehabilitation, TUQ, usability, UTAUT2

## Abstract

**Introduction:**

The use of telerehabilitation in the field of medical rehabilitation is increasing, particularly following the coronavirus pandemic, which accelerated the digitization of rehabilitation services. Patient acceptance is crucial for the sustainable implementation of these digital services. While most studies in the rehabilitation context focus on *behavioral intention* (attitudinal acceptance), this study additionally examines factors influencing *use behavior* (behavioral acceptance) in a real-world care setting. It also investigated patients’ usability experience and affinity for technology.

**Methods:**

A cross-sectional design was applied, recruiting participants from thirteen rehabilitation centers. Patients could participate in the survey digitally or via paper. Acceptance was measured based on the UTAUT2 model, supplemented with questions on *privacy concerns*, *usability*, and *technology affinity*. Data was first descriptively analyzed, followed by structural equation modeling to determine factors influencing *behavioral intention* and *use behavior*.

**Results:**

The analysis included 230 cases. Participants’ affinity for technology was in the medium range (M = 3.45, SD = 1.17; 1 = strongly disagree, 6 = strongly agree), while acceptance of telerehabilitation could be classified as high (M = 2.37, SD = 1.36; 1 = strongly agree, 7 = strongly disagree). The evaluation of the UTAUT2 model showed that the constructs *performance expectancy* (*p* < 0.001), *habit* (*p* < 0.001) and *hedonic motivation* (*p* < 0.001) had a significant positive impact on *behavioral intention*, while *habit* was the only construct that significantly influenced *use behavior* (*p* = 0.031). The model showed good explanatory power (*R*^2^ = 0.746) for *behavioral intention* but limited explanatory power for *use behavior* (*R*^2^ = 0.049). The constructs *habit* (*f^2^* = 0.117), *hedonic motivation* (*f^2^* = 0.085) and *performance expectancy* (*f^2^* = 0.120) contributed significantly to the predictive quality of the model.

**Discussion:**

Only a few constructs of the UTAUT2 model, namely *performance expectancy*, *hedonic motivation* and *habit, significantly* influenced *behavioral intention* in the German telerehabilitation context. Our results also showed that the hypothesized relationship between *behavioral intention* and *use behavior* was not statistically supported. This finding may reflect the “intention-behavior gap” described in the literature, in which behavior intentions do not necessarily lead to actual use.

## 1 Introduction

Digitization efforts in the field of telehealth and also in the specific field of telerehabilitation have increased significantly worldwide in recent years (1, 2). In Germany, too, various rehabilitation and especially aftercare services have recently been partially or fully digitized. This has been driven by the COVID-19 pandemic, which has led to the temporary or permanent closure of rehabilitation centers due to restrictive hygiene requirements and/or the cancellation of elective procedures (3, 4). One solution was to provide telerehabilitation, enabling rehabilitation and aftercare services to be provided across geographical distances while minimizing the risk of infection (5, 6). In Germany, some of these services continued after the pandemic and became part of standard care, while others were only temporarily authorized (7). Telerehabilitation here refers to “medical rehabilitation services delivered using information and communication technologies (ICT), i.e., assessment, monitoring, prevention, intervention, supervision, education, consultations, and counseling” (8). This includes digital services provided on-site in rehabilitation facilities to complement analog treatment.

The benefits of telehealth and the specific field of telerehabilitation services had already been known before the pandemic (9), but users were rather reluctant to use this service. The lack of acceptance, combined with insufficient usability of telehealth were seen as barriers to the implementation and dissemination of telehealth (10) and the specific field of telerehabilitation (11).

Users of telerehabilitation comprise both patients and healthcare professionals. Taking their respective needs into account is an important aspect of implementing telerehabilitation successfully (12). Studies on telerehabilitation acceptance conducted prior to the COVID-19 pandemic were largely carried out at a time when such services were not yet available to patients (11, 13, 14). Consequently, they primarily assessed hypothetical attitudes and behavioral intentions toward telerehabilitation rather than experiences based on actual use. Following the pandemic, more studies were conducted to examine telerehabilitation services after their introduction. Their findings indicate a growing openness toward and acceptance of these services overall (15, 16).

However, since the understanding of telerehabilitation varies greatly in international comparison, no conclusions could be drawn so far about the acceptance and use of telerehabilitation services specifically in Germany (17). Although some studies have recently examined telerehabilitation following actual use in Germany, these studies have mostly focused on specific systems, such as exergames (18), which have not yet become part of routine care. In line with technology acceptance research, a distinction can be made between *behavioral intention* (attitudinal acceptance), reflecting the individual's intention to use a technology, and *use behavior* (behavioral acceptance), reflecting the actual use of technology (19–21). Our study therefore aims to contribute to the understanding of technology acceptance in the German rehabilitation context under real-world conditions. A particular focus is placed on examining actual *use behavior* and the factors influencing it - aspects that are still insufficiently addressed in many studies (22, 23).

The aim of this study was to examine patients’ acceptance of telerehabilitation and to identify which factors contribute to a high level of acceptance. The focus was deliberately on the patient perspective, as the viewpoint of healthcare professionals regarding the implementation of telerehabilitation has already been explored in other parts of our research project and in previous studies (16, 24). In addition, factors such as usability and affinity for technology were taken into account.

To this end, we developed a structural equation model based on the Unified Theory of Acceptance and Use of Technology 2 (UTAUT2) to reveal which dimensions of technology acceptance influence both the *behavioral intention* and the *use behavior* of telerehabilitation. We have chosen the UTAUT2 model as the theoretical basis, as it is considered reliable for examining the acceptance of new technologies (20) and has also proven itself in the field of telehealth. Since the original work of Venkatesh et al. (21) is based on Partial Least Squares Structural Equation Modeling (PLS-SEM), the same methodology has been adopted in this study. Structural equation modeling is a well-established method for analyzing the determinants of technology acceptance (21, 25).

In addition, we have included questions about the participants’ affinity for technology and the usability of the telerehabilitation services, which were evaluated descriptively, as these are important factors which influence the implementation of telerehabilitation (26).

## 2 Theoretical framework and hypotheses

The theoretical framework for this study is UTAUT2, a widely recognized and reliable model for investigating the acceptance of new technologies. The UTAUT model was developed to combine the multitude of existing technology acceptance models into a single model. It is based on eight technology acceptance models, including *TAM*, *TAM2*, *the Theory of Reasoned Action* and the *Diffusion of Innovations Theory*. UTAUT2 builds on this foundation by enabling a more nuanced analysis of the factors influencing individuals’ acceptance and use of technology (20, 27).

The original version of UTAUT was developed to analyze the acceptance and use of technology in organizational contexts (20). Venkatesh et al. expanded the model in 2012 to adapt it to the consumer use context (known as UTAUT2) (21), and meanwhile it has also been applied in the field of e-health (28, 29). In our study, we apply the model to the more specific context of rehabilitation in Germany.

UTAUT2 includes seven constructs which potentially influence the *behavioral intention* of telerehabilitation: *performance expectancy, effort expectancy, social influence, facilitating conditions, habit, hedonic motivation* and *price value*. Since the aspect of data protection is becoming increasingly important, an additional dimension *privacy concerns* was included in the model, as in other previous studies (9, 26). Furthermore, the *price value* aspect was removed because the use of telerehabilitation in Germany is usually free of charge for the patients and covered mainly by the German pension insurance (9).

**Figure 1:**
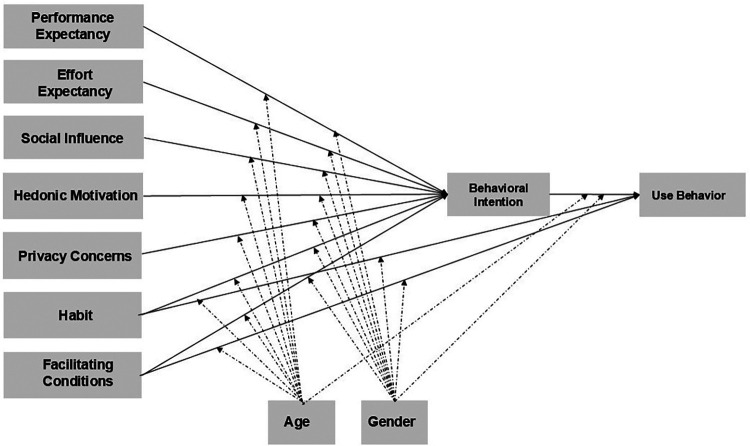
UTAUT2 research model adapted and extended for this study, based on Venkatesh et al. (21).

Three of the constructs directly influence *use behavior* in the model: *facilitation conditions, habit,* and *behavioral intention.* Venkatesh et al. supplemented the model with moderating variables such as *age*, *gender*, and *experience*. We omitted the aspect of *experience* because all our participants already had experience with telerehabilitation, but the time frame was too short to make any further meaningful subdivisions. We included the influence of *age* and *gender* in our hypothesis formation, knowing that the necessary prerequisites for these analyses could only be verified in the course of the multi-group analysis. Figure 1 shows the UTAUT2 research model as adapted for our study. The constructs and derived hypotheses included in our research model are presented in the following section.

### 2.1 Performance expectancy

In the original version of the UTAUT*, performance expectancy* is defined as “*the degree to which an individual believes that using the system will help them to attain gains in job performance”* (20). This has been transferred to the health sector by various authors (11, 30, 31) and was further refined by the authors of this study to fit the context of rehabilitation. In this setting *performance expectancy* is understood as *“the degree to which an individual believes that using telerehabilitation will help them*”. Based on this definition and its proven relevance in the field of technology acceptance research, it is theoretically assumed that *performance expectancy* may influence individuals’ *behavioral intention* to use telerehabilitation. Accordingly, the following hypothesis was formulated:

H1: *Performance expectancy* has a significant positive influence on the *behavioral intention* to use telerehabilitation.

### 2.2 Effort expectancy

According to the original UTAUT model, *effort expectancy* is defined as “*the degree of ease associated with the use of the system*” (20). In various studies, the original items of Venkatesh have been adapted for use in the health sector (11, 25, 26, 30, 31) and were further modified by the authors to fit the specific context of rehabilitation. In our study *effort expectancy* is defined as “*the degree of ease associated with the use of telerehabilitation”.* Based on the theoretical significance of *effort expectancy* in technology acceptance models, we assumed that *effort expectancy* influences individuals’ *behavioral intention* to use telerehabilitation. Accordingly, we proposed the following hypothesis:

H2: *Effort expectancy* has a significant positive influence on the *behavioral intention* to use telerehabilitation.

### 2.3 Social influence

According to the original UTAUT model, *social influence* is “*the degree to which an individual perceives that important others believe he or she should use the new system*” (20). Accordingly, we adapted the definition of *social influence* for this study as follows: “*Social influence is defined as the degree to which an individual perceives that important others, typically including family members, fellow patients, and healthcare professionals such as therapists, believe they should use telerehabilitation”*. Given the potential impact of *social influence* on *behavioral intention*, we assumed that an individual's perception of others’ opinions may impact their *behavioral intention* to use telerehabilitation.

We developed the following hypothesis:

H3: *Social Influence* has a significant influence on the *behavioral intention* to use telerehabilitation.

### 2.4 Facilitating conditions

According to the original UTAUT model facilitating conditions are defined as *“the degree to which an individual believes that an organizational and technical infrastructure exists to support use of the system*” (20). In the context of telerehabilitation, *facilitating conditions* refer not only to the availability of the necessary (technical) equipment required for the implementation, but also to the existence of sufficient knowledge and support to enable its use. In this study, *facilitating conditions* are defined as “*the degree to which an individual believes that an organizational and technical infrastructure exists to support use of telerehabilitation*”. Based on this concept, we proposed that *facilitating conditions* can play a decisive role in both the *behavioral intention* and the *use behavior* of telerehabilitation. Therefore, we formulated the following hypotheses:

H4: *Facilitating conditions* have a significant positive effect on the *behavioral intention* towards telerehabilitation.
H5: *Facilitating conditions* have a direct and significant positive influence on the *use behavior* of telerehabilitation.

### 2.5 Habit

The construct of *habit* was introduced in the extended version of the UTAUT as a determinant of user acceptance. It is defined as “*the extent to which people tend to perform behaviors automatically because of learning*” (21, 32). The definition has been adopted without modification for the context of telerehabilitation. Given the potential influence of *habit* on user acceptance, it is hypothesized that *habit* affects both the *behavioral intention* and the *use behavior* of telerehabilitation. Accordingly, we formulated the following hypotheses:

H6: *Habit* has a significant positive influence on the *behavioral intention* to use telerehabilitation.
H7: *Habit* has a direct and significant positive influence on the *use behavior* of telerehabilitation.

### 2.6 Hedonic motivation

The construct of *hedonic motivation* was also introduced in the extended version of the UTAUT model as a determinant of user acceptance. It is defined as “*the fun or pleasure derived from using a technology*” (21). The definition has been adopted without modification for the context of telerehabilitation. Due to the potential impact of *hedonic motivation* on the acceptance of telerehabilitation, we assumed that enjoyment associated with telerehabilitation use may influence individuals’ *behavioral intention* to adopt it. Therefore, the following hypothesis is proposed:

H8: *Hedonic motivation* has a significant positive influence on the *behavioral intention* of telerehabilitation.

### 2.7 Privacy concerns

*Privacy concerns* is a construct which is not part of the original UTAUT model. It is defined as “*the fear that personal data will not be adequately protected”*. This includes concerns that personal data will be misused and shared for unforeseeable purposes (33). This definition is directly applicable to the context of telerehabilitation. Considering the growing importance of data privacy in telehealth, as already mentioned, we assumed that *privacy concerns* may influence individuals’ *behavioral intention* to use telerehabilitation. Accordingly, we proposed the following hypothesis:

H9: *Privacy concerns* have a significant positive influence on the *behavioral intention* to use telerehabilitation.

### 2.8 Behavioral intention

*Behavioral intention* refers to the subjective probability that a person will perform a specific behavior (34), in our case, the use of telerehabilitation. As already mentioned, *behavioral intention* is widely recognized as a key predictor of *use behavior* in technology acceptance models. Therefore, we formulated the following hypothesis:

H10: *Behavioral intention* has a direct and significant positive influence on the *use behavior* of telerehabilitation.

### 2.9 Moderator variables

Building upon hypotheses H1 to H10, it is further assumed that these relations may be influenced by the moderator variables *age* and *gender*. Therefore, we proposed the following additional hypotheses:

H11: The relations assumed in H1 to H10 are moderated by the variable *age*.
H12: The relations assumed in H1 to H10 are moderated by the variable *gender*.

## 3 Materials and methods

### 3.1 Questionnaire

The questionnaire was developed so that it could be completed both online and as a paper-pencil version. It consists of a total of five sections:

First, general information on the use of telerehabilitation was collected using a total of 12 questions. The questions focused on the type of telerehabilitation, the context and purpose of use, the devices used, and the duration and frequency of use. Participants were also asked for any discontinuation of telerehabilitation use and the reasons behind it. The scale measuring frequency of use is based on the Media and Technology Usage and Attitudes Scale (35). This scale was previously defined by other authors as *use behavior* in the UTAUT2 and translated into German (36). For the purposes of this study, the response option “never” was excluded, as all participants who completed this section must have used telerehabilitation at least once. Additionally, the response options “once per hour,” “several times per hour,” and “constantly” were omitted, as these response options seem implausible in the context of telerehabilitation.

The second part of the questionnaire focused on the participants’ acceptance of telerehabilitation and refers to the UTAUT2 model. It contains 25 questions. Each construct of the UTAUT2 model was measured using three items, apart from *privacy concerns*, which were assessed using four questions. The items were adapted to the rehabilitation context based on previous studies in the field of telehealth. Where translation into German was required, the team approach was applied (37). As the survey was conducted while the digital services were already in use, the original hypothetical phrasing using “would” was reformulated in the present tense. All items were assessed using a 7-point Likert scale ranging from 1 (“strongly agree”) to 7 (“strongly disagree”).

The third part of the questionnaire dealt with further aspects of *usability* that had not yet been covered in the previous section. This section is based on the Telehealth Usability Questionnaire (TUQ) (38) and contains 10 Items. The TUQ normally consists of 21 items mapped across six dimensions: *usefulness*, *ease of use and learnability*, *interface quality*, *interaction quality*, *reliability*, and *satisfaction and future use* (38). We adapted it to this study. Since the *ease of use and learnability* dimension is included in the UTAUT *effort expectancy* scale, it was not surveyed again. The same applies to individual items of the *interface quality* dimension. The *interaction quality*-scale was not used because not all telerehabilitation services used in this study involved direct user interaction. Therefore, the analyses focused on the remaining dimensions *usefulness*, *reliability*, and *satisfaction and future use*, as they were expected to provide additional insights. Each of them was measured by three items and evaluated descriptively. These results should be viewed as a supplement to the UTAUT results. The authors translated the questions, which were originally developed for the broader context of telehealth, into German and adapted them for the specific context of telerehabilitation. Again, all items were assessed using a 7-point Likert scale ranging from 1 (“strongly agree”) to 7 (“strongly disagree”).

In the fourth section of the questionnaire, the participants’ affinity for technology was assessed using the Ultra Short Scale for Assessing Affinity for Technology Interaction in User Studies (ATI-S) (39). This brief version consists of only four items and was selected to keep the questionnaire concise and avoid overwhelming participants. The questions were measured using a 6-point Likert scale ranging from 1 (“strongly disagree”) to 6 (“strongly agree”).

The final section of the questionnaire included seven sociodemographic questions, such as age, gender, diagnosis group and level of education. Respondents were also asked to indicate whether they had completed the questionnaire independently or with assistance.

Important aspects of the questionnaire, along with the corresponding sub-scales and authors, can be found in Table 1*.* The questionnaire was tested for technical functionality (e.g., filter questions) and plausibility by eleven individuals using the online version on LimeSurvey (version 5.6.0) and by five people using the paper-and-pencil version. The pretest involved rehabilitation patients at a rehabilitation center as well as rehabilitation experts, who checked the questions for plausibility. Based on their feedback, the questionnaire was subsequently revised.

**Table 1:** Questionnaire items adapted to the rehabilitation context.

Questionnaire section	Constructs	Items	References
**Acceptance** **(UTAUT2)**	**Use Behavior**	How often do you use telerehabilitation?	(35, 36)
	**Performance Expectancy**	Using telerehabilitation increases the effectiveness of my treatment.	(30, 31)
		Using telerehabilitation is beneficial to my rehabilitation process.	(30, 31)
		Overall, telerehabilitation helps me to cope with my health problems.	(11, 30, 31, 61)
	**Effort Expectancy**	Learning how to use telerehabilitation is easy for me.	(25, 26, 36, 61)
		Interacting with telerehabilitation clear and understandable.	(25, 26, 36, 61)
		I find it easy to use telerehabilitation.	(25, 26, 36)
	**Social Influence**	People close to me recommend using telerehabilitation.	(11, 20, 26, 30, 31, 61)
		People whose opinions I value like me to use telerehabilitation.	(25)
		My therapist recommends me to use telerehabilitation.	(11, 30, 31)
	**Facilitating Conditions**	I do have all the necessary technical preconditions for using telerehabilitation.	(20, 30, 31, 61)
		I have the necessary knowledge to use telerehabilitation.	(25, 36, 61)
		I can get help from others when I have difficulties in using telerehabilitation.	(25, 36, 61)
	**Hedonic Motivation**	Using telerehabilitation is fun.	(25)
		Using telerehabilitation is enjoyable.	(25)
		I like using telerehabilitation.	(38)
	**Habit**	The use of telerehabilitation has become a habit for me.	(25, 62)
		The use of telerehabilitation is a matter of course for me.	(62)
		I believe that telerehabilitation has become an important part of my rehabilitation.	Own derivation based on original UTAUT
	**Behavioral Intention**	I can imagine continuing using telerehabilitation.	(25, 30)
		I can imagine using telerehabilitation regularly, if offered to me.	(11, 30, 61)
		I will recommend using telerehabilitation.	(30)
	**Privacy Concerns**	I am concerned that the information I disclosed to the service provider could be misused.	(33)
		I am concerned that a person can find private information about me.	(33)
		I am concerned about providing personal information to the service provider, because it could be used in a way I did not foresee.	(33)
		I am concerned about providing personal information to the service provider, because of what others might do with it.	(33)
**Usability** **(TUQ)**	**Usefulness**	Telerehabilitation improves my access to rehabilitation services.	(38)
		Telerehabilitation saves me time.	
		Telerehabilitation provides for my healthcare needs.	
	**Interface Quality**	This system is able to do everything I would want it to be able to do.	
	**Reliability**	I think telerehabilitation is equivalent to an analog service.	
		Whenever I make a mistake using the system, I can recover easily and quickly.	
		The system gives error messages that clearly tell me how to fix problems.	
	**Satisfaction and Future Use**	I feel comfortable using the telerehabilitation system.	
		Telerehabilitation is an acceptable way to receive healthcare services.	
		Overall, I am satisfied with this telerehabilitation system.	
**Affinity for Technology (ATI-S)**		I like to occupy myself in greater detail with technical systems.	(39)
		I like testing the functions of new technical systems.	
		It is enough for me that a technical system works; I don't care how or why.	
		It is enough for me to know the basic functions of a technical system.	

### 3.2 Data collecting process

#### 3.2.1 Sample size

The tenfold rule is often used to estimate the minimum sample size in PLS-SEM. Accordingly, the sample size should be ten times the highest number of formative indicators used to measure a single construct, or ten times the highest number of structural paths directed at a specific construct in the structural model (40). For our calculations, this would require a sample size of 70. However, recent literature recommends performing statistical analyses to determine the sample size, as these analyses provide more reliable information (41). Using G*Power (version 3.1.9.7) and a medium effect size (*f^2^* = 0.15), a significance level of *α* = 0.05, a recommended power of 0.80, and up to seven predictors in the structural model, the required minimum sample size was estimated as *n* = 103. This ensures sufficient power to reliably detect significant effects (41, 42).

#### 3.2.2 Recruitment

All rehabilitation centers in Germany which offer telerehabilitation services were defined as potential sources for study participants. The study population included all rehabilitation patients at these facilities who were aged at least 18 at the time of the survey and had used telerehabilitation within the last six months, regardless of their diagnosis.

For the recruitment, all homepages of inpatient rehabilitation centers were searched for digital services using the “Directory of hospitals and preventive care/rehabilitation facilities in Germany“. If digital services were available, their publicly accessible email address was added to a mailing list. We were therefore able to request the support of 159 institutions for the survey and make the survey documents available to their patients. In addition, the “Federal Association of Outpatient Medical Rehabilitation Centers“ was contacted and asked to draw the attention of outpatient rehabilitation centers to the survey and to ask them to participate in the survey if they were using telerehabilitation. The number of outpatient facilities reached by the email distribution list is not known here. Thirteen rehabilitation centers ultimately contacted us and agreed to forward the survey documents to their patients.

#### 3.2.3 Conducting the survey

The survey was conducted as a hybrid survey. Participation was possible both online via a survey platform (LimeSurvey, version 5.6.14) and in print version. Posters were put up in the rehabilitation centers to inform people about the digital participation option. The questionnaire could be accessed via a QR code. Patients who wanted to participate from home – for example, those in an aftercare program – received a link and QR code to the survey by email. For analogous participation, the survey material, including a pre-stamped envelope, was sent to the rehabilitation centers where the staff handed out the questionnaires accordingly. The survey period was from 02 May 2023 to 01 Aug 2023.

### 3.3 Data cleaning and descriptive analysis

After the survey was completed, the data collected via LimeSurvey was exported to SPSS and cleaned. Paper-based questionnaires were manually entered into SPSS. A total of 291 individuals accessed the questionnaire. When preparing the data for PLS-SEM, it is recommended to exclude cases from the dataset if more than 15% of the values for that case are missing (40). We applied this 15% rule to the entire data set. In view of this, 54 cases were initially excluded. Of these, 31 answered fewer than three questions. An additional seven cases could not be analyzed due to inconsistent responses. Thus, 230 cases were included in the analysis. Of these, 193 were answered digitally and 37 were answered in analog form.

After data cleaning, a descriptive analysis of technology acceptance, usability, and technology affinity was conducted in SPSS. A few cases were missing for individual items included in the descriptive analysis of the sociodemographic data. This is noted in the presentation of the results.

### 3.4 Structural equation modeling

To identify the drivers of technology acceptance, structural equation modeling was performed using SmartPLS 4. Following the approach of Hair et al. (41), the analysis proceeded in two steps: First, the measurement model was evaluated, second the structural model was estimated. Since all cases contained complete data for the included variables, no further action was required regarding missing values.

#### 3.4.1 Evaluation of the measurement model

All constructs of the measurement model were specified reflectively, except *use behavior*, which was modeled as a single-item construct. Each construct represented a latent variable operationalized by three to four items. Given that the data deviated from normality, the use of PLS-SEM was appropriate, as it is a non-parametric approach which does not require normally distributed data.

Reliability and validity were assessed as part of the measurement model evaluation. *Reliability* was examined at the indicator level (indicator reliability) and at the construct level (internal consistency reliability). Indicator reliability was assessed using the outer loadings of the indicators, which should be 0.708 or higher. Internal consistency reliability, measured via composite reliability and Cronbach's alpha, should be between 0.70 and 0.950 (41).

Convergent and discriminant validity were examined to confirm the validity of the measurement model. The key criterion *for convergent validity* is *the average variance extracted* (AVE), which should be above the threshold of 0.50. This indicates that the respective construct explains more than half of the variance of its indicators (41). Discriminant validity indicates the extent to which a construct is empirically independent, i.e., the extent to which it differs from the other constructs. To assess discriminant validity, the heterotrait-monotrait ratio (HTMT) was used. Recommended thresholds are 0.9 for conceptually similar constructs and 0.85 for conceptually distinct constructs. To ensure that the HTMT values were significantly below the threshold, a bootstrapping procedure with 10,000 subsamples was performed to calculate the 95% bootstrapped confidence intervals for the HTMT values (41, 43).

#### 3.4.2 Evaluation of the structural model

The evaluation of the structural model included an examination of the relationships between the constructs as well as the model's predictive power. Various methods proposed by Hair et al. (41) were applied for this purpose. These included examining collinearity (via Variance Inflation Factor), the relevance and significance of the path coefficients, explanatory power (*R^2^* values), and predictive power (PLSpredict and CVPAT) (41).

The Variance Inflation Factor (VIF) measures the degree of collinearity among the independent variables. High multicollinearity can hinder the accurate estimation of each variable's influence. Ideally, VIF values should be below three, though values up to five are acceptable. Path coefficients (*β*) represent the relationships between constructs. They are standardized and typically range from −1 to +1. A value close to +1 indicates a strong positive relationship, while a value close to −1 indicates a negative relationship. The closer the estimated value is to 0, the weaker the relationship between the constructs. *p*-values are used to test significance. At a commonly used significance level of 5%, a *p*-value less than 0.05 indicates a statistically significant relationship. The *R^2^* value is the most commonly used coefficient to calculate the explanatory power of a model. *R^2^* values should be between 0 and 1; the higher the value, the greater the explanatory power (41).

The *f^2^* effect sizes indicate “whether an exogenous construct has a substantial influence on an endogenous construct” (41). They show which constructs contribute most to the *R*^2^ value. According to commonly used thresholds, a *f^2^* value of 0.02 indicates a small effect, 0.15 indicates a medium effect and 0.35 indicates a large effect (41).

To assess the predictive power of a model, Hair et al. (41) recommend applying the PLSpredict procedure. The predictive power refers to the extent to which the model can accurately predict values for observations that were not used to estimate the model, i.e., out-of-sample (41, 44). The evaluation was based on Q^2^_predict_ and the root mean square error (RMSE). To demonstrate a strong predictive power of the model, two conditions should be met: Q^2^_predict_ must be greater than zero and the RMSE value of each indicator variable of the considered endogenous construct must be less than the RMSE value of the corresponding construct of a linear comparison model (41, 44).

Hair et al. (41) also recommend carrying out the Cross-Validated Predictive Ability Test (CVPAT) as described by Liengaard et al. (45) and extended by Sharma et al. (46) to determine whether the predictive power of the present PLS path model at the construct level is significantly higher than that of a comparative model. Therefore, the PLS-SEM results were compared with the loss values of the indicator average (IA) and the linear model (LM). If the PLS-SEM shows a negative average loss difference compared to the IA or LM, and the *p-*value is below the defined significance threshold of 0.05, the model is considered to have significantly better predictive power than the benchmark (41).

#### 3.4.3 Multi-group analysis

As outlined in the introduction, conducting a multi-group analysis (MGA) is recommended, particularly to examine sociodemographic differences. This is because neglecting possible heterogeneity can lead to misleading conclusions (47). According to Hair et al. (41), a MGA is the most efficient way to evaluate moderation effects across multiple relationships (41).

However, MGA requires prior verification of measurement invariance, using a three-step MICOM procedure (Measurement Invariance of Composite Models). If the conditions MICOM conditions are met, the MGA can be conducted in SmartPLS.

## 4 Descriptive results

### 4.1 Sociodemographic data

230 questionnaires were included in the analysis. One person stated that they had completed the questionnaire with assistance. Respondents ranged in age from 20 to 89 years, with the majority of respondents between the ages of 50 and 59 (*n* = 121, 52.6%). At 142 (61.7%), slightly more than half of the respondents were women. Most respondents were in the neurology indication group (*n* = 128, 55.7%), followed by psychosomatics/psychotherapy (*n* = 32, 13.9%), and orthopedics (*n* = 25, 10.9%). The complete sociodemographic data is shown in Table 2*.*

**Table 2:** Sociodemographic data of the participants.

Sociodemographic data	Indication group
Age groups (*n* = 230)	% (n)
20–29	3,9 (9)
30–39	8,3 (19)
40–49	15,7 (36)
50–59	52,6 (121)
60–69	17,8 (41)
80–89	0,4 (1)
I prefer not to answer	0,9 (2)
No response	0,4 (1)
Gender	% (n)
Male	36,5 (84)
Female	61,7 (142)
I prefer not to answer	1,3 (3)
No response	0,4 (1)
Indication group	% (n)
Addiction disorders	0,4 (1)
Cardiology	7,4 (17)
Neurology	55,7 (128)
Oncology	1,7 (4)
Orthopedics	10,9 (25)
Pulmonology	1,7 (4)
Psychosomatics/Psychotherapy	13,9 (32)
Metabolism/Internal medicine	0,4 (1)
I prefer not to answer	2,6 (6)
Other indications	3,0 (7)
No response	0,4 (1)
Not valid	1,7 (4)
Educational level	% (n)
Doctorate	1,7 (4)
Master's degree or equivalent educational program	6,5 (15)
Diploma	17,4 (40)
Bachelor's degree or equivalent educational program	14,8 (34)
Completed vocational training	28,7 (66)
General university entrance qualification (Abitur)	5,7 (13)
Technical college entrance qualification (Fachabitur)	6,5 (15)
Secondary school leaving certificate (Realschulabschluss and Hauptschulabschluss)	13,4 (31)
I prefer not to answer	3,9 (9)
Others	0,9 (2)
No response	0,4 (1)
Cost bearer	% (n)
Statutory pension insurance	88,7 (204)
Statutory health insurance	5,7 (13)
Private health insurance	2,2 (5)
I prefer not to answer	1,7 (4)
Others	0,9 (2)
No response	0,9 (2)

### 4.2 Types, purposes and frequency of telerehabilitation use

Most respondents (*n* = 185, 80.4%) used a telerehabilitation app, while 35 (15.2%) used an internet portal. Video conferencing programs (*n* = 2, 0.9%) or other telerehabilitation services (*n* = 8, 3.5%) were hardly reported.

The telerehabilitation services were mainly used on smartphones (*n* = 185, 80.4%), but also on tablets (*n* = 57, 24.8%), laptops/desktop computers (*n* = 52, 22.6%) and other screens (*n* = 24, 10.4%). Smartwatches and other devices played a minor role (*n* = 5, 2.2%). Multiple devices could be specified.

Telerehabilitation was mainly employed for exercising (*n* = 186, 80.9%), participation in training courses (*n* = 134, 58.3%) and information gathering (46.1%,). The services were used several times a week by 138 (60.0%) and once a week by 41 (17.8%) of the respondents. 20 (8.7%) used telerehabilitation once a day and 6 (2.6%) respondents used telerehabilitation several times a day. 4 (1.7%) tried telerehabilitation only once. Only 5 (2.2%) participants reported discontinuing the use of telerehabilitation. Most respondents (*n* = 165, 71.7%) made use of telerehabilitation as part of their rehabilitation aftercare. An overview of use purposes, devices used, and detailed information on use times and duration is provided in Additional File 1.

### 4.3 Affinity for technology

Participants’ technology affinity was measured using a Likert scale ranging from 1 to 6 (1 = low agreement, 6 = high agreement). The internal consistency of the scale was high (Cronbach's *α* = 0.839). The overall mean score of 3.45 (SD = 1.17) suggests a moderate level of technology affinity, with higher values corresponding to a stronger interest in technology.

### 4.4 Acceptance

As already described, the UTAUT2 model was used to investigate the acceptance of telerehabilitation. The *behavioral intention* dimension reflects the degree of attitudinal acceptance, whereas *use behavior* represents behavioral acceptance. Since *use behavior* was operationalized as frequency of use, the corresponding descriptive results have already been reported in Section 4.2. The remaining UTAUT2 constructs represent factors that may influence attitudinal and behavioral acceptance. All constructs were rated on a 7-point Likert scale (1 = strongly agree, 7 = strongly disagree). Accordingly, lower mean values indicate stronger agreement.

The quality criteria for the acceptance scales are reported in the course of structural equation modeling, which is why they are not included in the descriptive evaluation.

The results show that the *behavioral intention* was high, with a mean of 2.37 (SD = 1.36). Participants also rated the factors influencing acceptance positively. *Facilitating conditions*, which reflect the availability of a supportive infrastructure, were rated very positively (M = 1.64, SD = 0.68), closely followed by the *effort expectancy*, which includes the perceived ease of use (M = 1.70, SD = 0.70). *Hedonic motivation* (M = 2.3, SD = 1.3) and *performance expectancy* (M = 2.30, SD = 1.21) also scored well, showing that patients believe that telerehabilitation offers significant benefits and are motivated to use the services.

*Habit*, which indicates the development of use routines, received a more moderate rating (M = 2.76, SD = 1.38), suggesting that regular use has not yet become fully established among participants. The aspect of *social influence* also received a moderate rating (M = 3.12, SD = 1.36), meaning that the opinions of third parties do not play a major role in the use of telerehabilitation.

In contrast, the dimension *privacy concerns* received a relatively high mean value (M = 5.05, SD = 1.49), indicating that participants did not express strong concerns about data protection in the context of using telerehabilitation.

### 4.5 Usability

As in the acceptance measurements, all constructs were rated on a 7-point Likert scale (1 = strong agreement, 7 = strong disagreement). The internal consistency of the scales used was acceptable to high (Cronbach's *α*: *usefulness = 0.784*; *reliability = 0.704*; *satisfaction and future use = 0.869*). The evaluation of the usability dimensions revealed a rather positive perception among the participants. The telerehabilitation service was perceived as relatively *useful* for the rehabilitation process (M = 3.00, SD = 1.29). *Reliability* was rated more neutrally (M = 3.53, SD = 1.23), indicating that users neither clearly agreed with nor rejected the reliability of the service. *Satisfaction* with telerehabilitation received relatively good ratings (M = 2.55, SD = 1.20).

## 5 Results of structural equation modeling

### 5.1 Evaluation of the measurement model

#### 5.1.1 Reliability and validity

The measurement model met the quality criteria regarding indicator reliability and internal consistency reliability. As shown in Table 3, all outer loadings exceeded the recommended threshold of 0.708, meaning that indicator reliability was given.

**Table 3:** Indicator reliability, internal consistency reliability and convergent reliability.

Construct	Items	Indicator Reliability	Cronbachs Alpha	rho_a	AVE
Performance Expectancy	V13_AkzpPEEf	0.951	0.944	0.947	0.899
	V14_AkzpPEPr	0.960			
	V15_AkzpPEBe	0.933			
Effort Expectancy	V16_AkzpEEUm	0.835	0.825	0.827	0.741
	V17_AkzpEEVe	0.865			
	V18_AkzpEEBe	0.881			
Social Influence	V19_AkzpSIEm	0.853	0.762	0.760	0.682
	V20_AkzpSINu	0.886			
	V21_AkzpSITh	0.730			
Facilitating Conditions	V22_AkzpFCTe	0.891	0.744	0.746	0.666
	V23_AkzpFCKe	0.835			
	V24_AkzpFCHi	0.713			
Hedonic Motivation	V25_AkzpHMSp	0.963	0.958	0.959	0.922
	V26_AkzpHMAn	0.953			
	V27_AkzpHMGe	0.965			
Habit	V28_AkzpHAGe	0.912	0.913	0.916	0.852
	V29_AkzpHASe	0.943			
	V30_AkzpHaWi	0.914			
Behavioral Intention	V31_AkzpBIZu	0.970	0.927	0.930	0.873
	V32_AkzpBIRe	0.929			
	V33_AkzpBIEm	0.903			
Privacy Concern	V34_AkzpPCDm	0.934	0.959	0.983	0.889
	V35_AkzpPCPr	0.950			
	V36_AkzpPCSo	0.954			
	V37_AkzpPCZw	0.934			

The Internal consistency reliability, measured using composite reliability (*rho*_*a)* and Cronbach's Alpha was within the acceptable range of 0.7 to 0.950. For the constructs *performance expectancy* and *hedonic motivation* the internal consistency reliability slightly exceeded 0.95, which could indicate redundancy. However, as these constructs are well established in the field of e-health acceptance research, they have been retained unchanged (9, 20, 25, 48).

#### 5.1.2 Convergent and discriminant validity

The measurement model exhibited good convergent and discriminant validity. As shown in Table 4*,* all AVE values exceeded the recommended threshold of 0.50 and almost all HTMT values were below the stricter limit of 0.85, except for the construct pairs *habit* and *hedonic motivation* and *habit* and *behavioral intention*. As these constructs were conceptually related and the HTMT values remained below the threshold of 0.90, discriminant validity was considered to be given for all constructs. However, the upper limits of the 95% bias-corrected confidence intervals for the constructs were slightly above 0.9 (0.918 and 0.915), which indicated some uncertainty regarding discriminant validity. Therefore, discriminant validity was considered acceptable but should be interpreted with caution (49). Detailed HTMT bias-corrected confidence intervals are reported in Additional File 2*.*

**Table 4:** Discriminant validity (HTMT).

Construct	PE	EE	SI	FC	HM	HA	PC	BI	UB
**PE**									
**EE**	0.532								
**SI**	0.683	0.372							
**FC**	0.423	0.781	0.442						
**HM**	0.803	0.654	0.654	0.554					
**HA**	0.818	0.539	0.740	0.466	0.881				
**PC**	0.143	0.327	0.130	0.347	0.218	0.176			
**BI**	0.834	0.525	0.653	0.444	0.849	0.878	0.149		
**UB**	0.110	0.156	0.073	0.060	0.171	0.225	0.020	0.154	

PE: Performance Expectancy, EE: Effort Expectancy, SI: Social Influence, FC: Facilitating Conditions, HM: Hedonic Motivation, HA: Habit, PC: Privacy Concerns, BI: Behavioral intention, UB: Use Behavior.

### 5.2 Evaluation of the structural model

#### 5.2.1 Collinearity (VIF)

According to Table 5*,* all VIF values were below the acceptable limits of five. The highest value, 4.197, was found for the relationship between *hedonic motivation* and *behavioral intention*. Although this value was acceptable, it indicated a relatively high correlation and was therefore not ideal.

**Table 5:** Collinearity (VIF).

Constructs	VIF
Performance Expectancy – Behavioral intention	2.905
Effort Expectancy – Behavioral intention	1.994
Social Influence – Behavioral intention	1.754
Facilitating Conditions – Behavioral intention	1.708
Hedonic Motivation – Behavioral intention	4.197
Habit – Behavioral intention	3.876
Privacy Concerns – Behavioral intention	1.125
Behavioral intention – Use Behavior	2.960
Facilitating Conditions – Use Behavior	1.188
Habit – Use Behavior	2.989

#### 5.2.2 Relationships between the constructs based on the path coefficients and *p*-values

**Figure 2:**
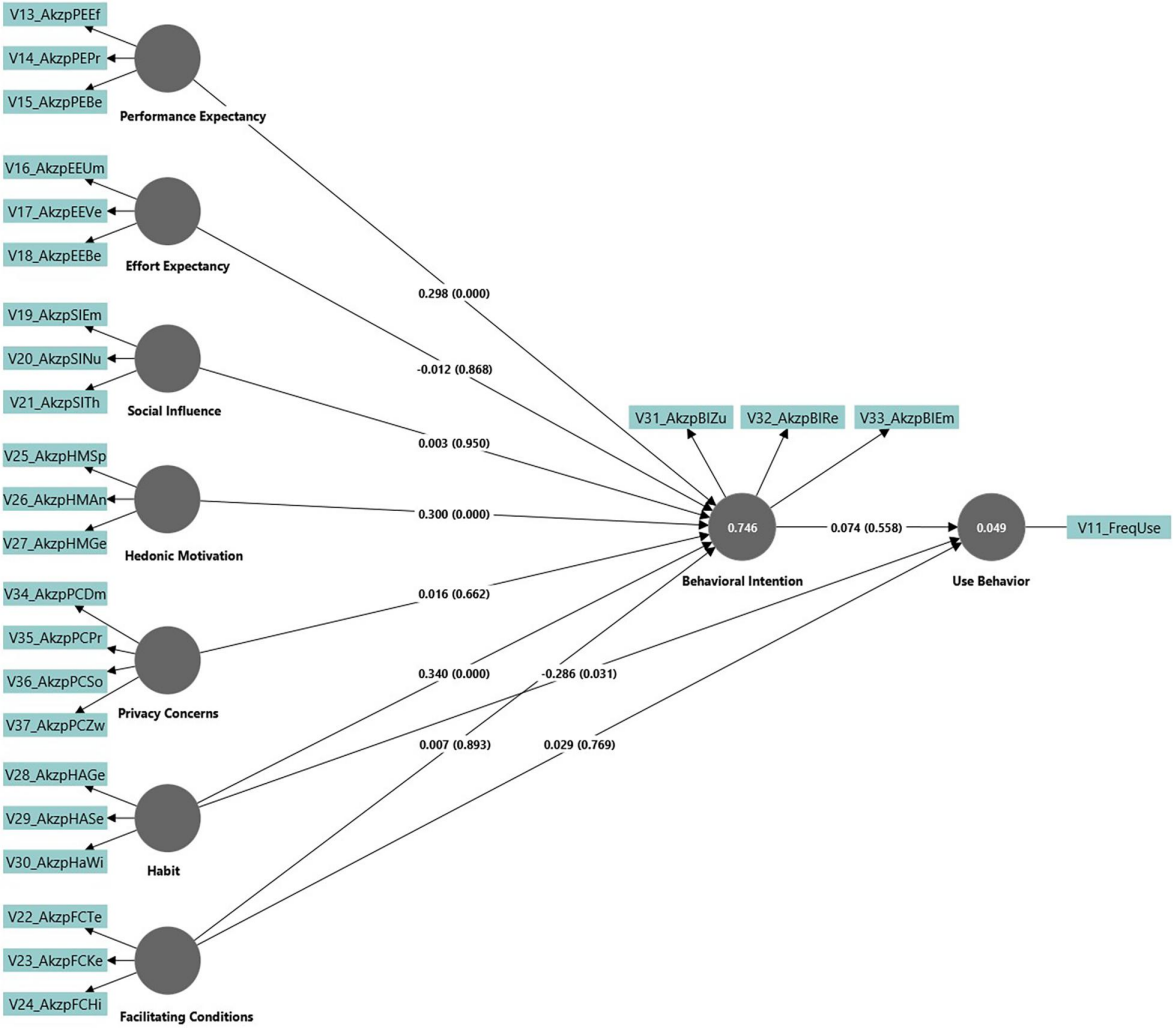
Path coefficients.

 Figure 2 shows the path coefficients and *p*-values of our model. As can be seen, *habit (β* *=* 0.340, *p* *<* *0.001*)*, hedonic motivation (β* = 0.300, *p* < 0.001*)* and *performance expectancy (β* = 0.298*, p* < 0.001*)* each had a significant positive influence on *behavioral intention*, while the other constructs did not. This indicates that higher levels of *motivation, performance expectancy*, and *habit* regarding telerehabilitation, were associated with higher *behavioral intention*. In terms of *use behavior*, only *habit* showed a significant effect, however, this relationship was negative (*β* = −0.286, *p* = 0.031). In other words, higher ratings on the *habit* construct, were associated with lower level of use. This unexpected result seems implausible at first and will be explored in the discussion.

The calculation of the bias-corrected confidence intervals, detailed in Additional File 2, confirmed the statistical significance of the results.

In summary, hypotheses H_1_, H_6_, and H_8_ were supported by the data, while H_2_, H_3_, H_4_, H_5_, H_7_, H_9_, and H_10_ were rejected.

#### 5.2.3 Explanatory power of the model (R^2^ values and f^2^ effect sizes)

As shown in Figure 2 *behavioral intention*, with an *R^2^* value of 0.746, was well explained by the latent variables (constructs) in the structural model, while the *R^2^* value for *use behavior* was close to zero (*R^2^* = 0.049) and was not adequately explained by the latent variables. This means that 95 percent of the variance was explained by other variables that were not included in the model.

With regard to effect sizes, *habit* (*f^2^* = 0.117), *performance expectancy*(*f^2^* = 0.120) and *hedonic motivation* (*f^2^* = 0.085) had a minor influence on *behavioral intention*, while *habit* also showed a minor influence on *use behavior* (*f^2^* = 0.029). In contrast, *effort expectancy*, *social influence*, *facilitating conditions* and *privacy concerns* had no relevant effects on *behavioral intention*, just as *behavioral intention* and *facilitating conditions* had no effect on *use behavior*. Table 6 shows a comparison of all *f^2^* effect sizes.

**Table 6:** *f^2^* effect sizes.

Constructs	*f^2^* effect sizes
Performance Expectancy – Behavioral Intention	**0**.**120**
Effort Expectancy – Behavioral Intention	0.000
Social Influence – Behavioral Intention	0.000
Facilitating Conditions – Behavioral Intention	0.000
Hedonic Motivation – Behavioral Intention	**0**.**085**
Habit – Behavioral Intention	**0**.**117**
Privacy Concerns – Behavioral Intention	0.001
Behavioral Intention – Use Behavior	0.002
Facilitating Conditions – Use Behavior	0.001
Habit – Use Behavior	**0**.**029**

#### 5.2.4 Predictive power of the model

As shown in Table 7*,* the evaluation of Q^2^_predict_ and RMSE indicated that the model has good predictive power, since both conditions are met: Q^2^_predict_ values were greater than zero, and the RMSE of each indicator of the considered endogenous construct was lower than that of the corresponding indicator of a linear comparison model.

**Table 7:** Q^2^predict and RMSE values.

Indicators	Q^2^predict	PLS-SEM_RMSE	LM_RMSE
V31_AkzpBIZu	0.669	0.838	0.874
V32_AkzpBIRe	0.527	1.089	1.119
V33_AkzpBIEm	0.683	0.746	0.766
V11_FreqUse	0.014	0.972	1.050

However, as Tables 8, 9 demonstrate, while both benchmarks exhibited a negative loss difference, the outcome for *use behavior* was not statistically significant compared to the IA (*p* = 0.697), and likewise, the outcome for *behavioral intention* was not statistically significant compared to the LM *(p* = 0.088). Therefore, in both cases, the predictive power of the SEM was not significantly better than that of the prediction benchmark. Nevertheless, when considering the results of both prediction benchmarks overall, the overall predictive power of the model could still be considered satisfactory (*p* < 0.001 and *p* = 0.003).

**Table 8:** PLS-SEM vs. Indicator Average.

Construct	Average loss difference	T-value	*p*-value
**Behavioral Intention**	−1.312	5.928	< 0,001
**Use Behavior**	−0.013	0.390	0.697
**Overall**	−0.988	5.957	< 0,001

**Table 9:** PLS-SEM vs. Linear Model.

Construct	Average loss difference	T-value	*p*-value
**Behavioral Intention**	−0.054	1.714	0.088
**Use Behavior**	−0.159	3.134	0.002
**Overall**	−0.080	3.016	0.003

### 5.3 Multi-group analysis

The results described refer to the entire study population. In order to perform the multi-group analysis, the prerequisites were first checked using a three-stage MICOM analysis.

In our case, the second step, testing the compositional invariance, was not fulfilled for the moderator *gender*, specifically for the variable *behavior intention* variable, because the permutation *p*-value was 0.044 and thus below the commonly accepted threshold of 0.05 (for additional values from the MICOM analysis, please refer to Additional File 3*)*. This indicated a significant difference between the groups, meaning that compositional invariance was not established for this construct. Therefore, it was not possible to interpret the group differences here comparatively as an MGA could have led to misleading or incorrect results.

Concerning the moderator *age*, we decided after descriptive analysis not to perform an MGA due to the unequal group sizes, as a mismatch in group sizes could have led to distorted results (47, 50).

Since the prerequisites for the moderator analysis could not be met, hypotheses H11 and H12 could neither be confirmed nor rejected.

## 6 Discussion

The aim of our study was to describe the usability, acceptance, and affinity for technology of rehabilitation patients and to investigate the factors that influence the acceptance of telerehabilitation in medical rehabilitation in Germany. For this purpose, the acceptance model developed by Venkatesh et al. (21) was considered the most appropriate theoretical framework because it had already been used in related fields, such as e-health (9, 25, 28, 29, 48). This provided a good basis for comparing results. As other authors have done before, we modified the model to fit the context of telerehabilitation. Based on the literature, we also included the aspect of *privacy concerns* (25).

Our initial descriptive evaluation revealed predominantly positive results regarding the usability and attitudinal acceptance of telerehabilitation among participants. Although direct comparisons with previous studies are difficult due to differences in measurement methods and the technologies investigated, our results are consistent with earlier research reporting high patient satisfaction with telerehabilitation services (51, 52). At the same time, some studies have also described limitations regarding the reliability of these services (53).

The technological affinity of our participants was moderate, indicating a neutral to slightly positive attitude toward technology. While other studies in the field of e-health often assume that higher acceptance levels are driven by a self-selection bias, whereby individuals with a strong affinity for technology are more likely to participate (54), in our case, it can be assumed that the high level of acceptance is not solely attributable to the selective participation of particularly tech-savvy individuals. However, further analyses would be necessary to draw a more robust conclusion. For example, affinity for technology could be included in the UTAUT-model as an additional predictor of *behavioral intention* in future analyses.

The focus of our study was on identifying the factors that contribute to the *use intention* (attitudinal acceptance) and *use behavior* (behavioral acceptance) of Telerehabilitation. The results of the structure equation modeling showed that *performance expectancy*, *habit*, and *hedonic motivation* influence *behavioral intention* significantly. According to Venkatesh et al. (21), *performance expectancy* is a strong predictor for *behavioral intention*, a finding that has been confirmed by further studies (9, 20). In our model, *habit* was the strongest predictor for the *behavioral intention* (*β = *0.340, *p* < 0.001). A study in the related field of mHealth similarly identified *performance expectancy* and *habit* as the most influential drivers of behavioral intention (55). *Hedonic motivation* has also been described in studies from related fields as a predictor for *behavioral intention.* In one study, it was even the only one (9).

However, our hypothesis that *privacy concerns* might have a significant impact when added to the established dimensions was not confirmed. *Privacy concerns* did not significantly contribute to the *behavioral intention* of telerehabilitation.

Many of the acceptance studies conducted in the context of rehabilitation do not take *use behavior* (behavioral acceptance) into account and report only on *behavioral intention* as an indicator for attitudinal acceptance (22, 23). Therefore, our study aimed to extend existing research by examining *use behavior* and the factors that influence it. In Venkatesh's UTAUT2 model, *use behavior* is directly influenced by the constructs of *intention to use*, *habit*, and *facilitating conditions*. Our results revealed that *behavioral intention* did not significantly influence *use behavior*. This may seem surprising at first, but other studies in the field of e-health have also reported similar findings (55, 56). This phenomenon is known as the intention-behavior gap, and it indicates that *behavioral intention* does not necessarily result in actual use (57). It has been observed particularly in health-related studies that deal with physical activity. One possible explanation for this is that the scales used to measure *behavioral intention* and *use behavior* are not sufficiently aligned (58). This goes back to the considerations of Ajzen & Fishbein, who dealt extensively with attitude-behavior relations (59). In our study, for example, *use behavior* was assessed with specific frequency-based questions such as “once a day”, while *behavioral intention* was measured using more general terms like “regular use”.

Another explanation for the lack of correlation between *behavioral intention* and *use behavior* is that patients may have perceived telerehabilitation as helpful and indicated that they would use it again if needed, but this did not necessarily translate into actual use, which was likely influenced by other external or structural factors unknown to us.

Another notable finding was the significant negative impact of *habit* on *use behavior*. This result was unexpected and, to our knowledge, had not been found in previous studies. Various considerations are therefore needed to explain it: First, the model showed an additional indirect path from *habit* via *behavioral intention* to *use behavior* suggesting a possible mediation effect. However, since the path from *behavioral intention* to *use behavior* was neither strong nor statistically significant, there was no clear evidence for mediation. Looking at the two constructs – *habit* and *use behavior* – in isolation, the negative correlation remained. This suggests that there must be other reasons for this negative correlation. One possible explanation is that subjective routines or habits developed positively during use, thereby increasing the *behavioral intention*, while more intensive or frequent use may have been externally constrained by rehabilitation schedules or prescription-related limitations. However, such restrictions would typically lead to a weakened association, but not to a reverse association. The negative relationship between the two constructs therefore remains difficult to explain and should be the subject of further research.

Our study also examined the extent to which the constructs explained *behavioral intention* and *behavior*. The results showed that 75% of the variance in *behavioral intention* was explained by the structural model, with *performance expectancy, hedonic motivation,* and *habit*, contributing most strongly to this construct. However, the results also showed that only 5% of the variance in *use behavior* was explained by the associated constructs. This leaves several possibilities for interpretation: Either other, more influential constructs - such as knowledge or skills - affected *use behavior*, which should be examined in further studies. Alternatively, there is some evidence suggesting that the scale used to measure *use behavior* was not suitable for the rehabilitation context. The scale examined *use behavior* based on the assumption that the frequency of using a technology was freely selectable. However, in the case of telerehabilitation, this assumption applied only to a limited extent, as frequency of use was partly predetermined, e.g., when using apps, and externally limited, for example by prescriptions. However, this circumstance became apparent only during the qualitative interviews, which took place after the survey had been completed. Therefore, it could not be taken into account in this study.

Another aspect of the study that we wanted to examine was the moderator analysis. Unfortunately, due to the absence of the aforementioned conditions, this was not possible. Consequently, the study does not provide any insights into the extent to which predictors of *intention to use* differ in relation to sociodemographic variables. Nevertheless, it is essential for future studies to consider these potential differences between groups when designing the study.

The results highlighted the uncertainty of which factors most strongly influence actual *use behavior* regarding telerehabilitation. This suggests that the current model may not fully capture the determinants of this construct. Future studies should consider adapting the UTAUT framework for use in rehabilitation settings. Additionally, the operationalization of the construct should be reviewed, and more context-specific measurement instruments should be developed. It should be noted in particular that measuring instruments from related fields such as e-health cannot necessarily be adopted directly. Some of these applications, such as certain apps, can be used as often as desired. This is not the case in the rehabilitation sector and therefore represents a special circumstance that must be taken into account.

Overall, however, the study allows well-founded conclusions to be drawn about the factors that significantly influence the attitudinal acceptance of telerehabilitation services. When implementing these services, rehabilitation facilities should therefore pay particular attention to the factors of *habit*, *performance expectancy*, and *hedonic motivation*. Establishing use routines and strengthening patient motivation are particularly important in this regard. To strengthen use routines, for example, reminder functions could be expanded, and patients could be supported in integrating telerehabilitation measures into their daily lives, especially during follow-up care. Individualized patient feedback could help boost motivation, and gamification elements may also support sustained engagement. Furthermore, it is crucial to clearly communicate the benefits of telerehabilitation to patients in terms of their own health and rehabilitation process, as the results show that high *performance expectancy* is associated with a strong *behavioral intention* to use the service.

## 7 Limitations and strength of the study

Beyond the challenges already discussed regarding the operationalization and limited explanatory power of the *use behavior* construct, our study has limitations that restrict the representativeness of the results. A disproportionately high percentage of patients with neurological conditions participated in the survey, which does not accurately reflect the distribution of diagnoses in the field of rehabilitation (60). This is due to the self-selection of rehabilitation centers who agreed to support and participate in the study. Furthermore, due to the anonymous nature of the online survey, it cannot be entirely ruled out that some individuals participated multiple times. However, the data review provided no evidence of this. In addition, it would have been useful to conduct a longitudinal study to examine changes in acceptance over the period of use.

Despite these limitations, our study has several strengths. Extending previous research, our study examines factors influencing the *use behavior* of telerehabilitation services and provides important insights into the applicability of the UTAUT model in the rehabilitation context.

In addition to the known dimensions of the UTAUT scale, the study integrates the important aspect of data protection. Furthermore, the high explanatory power of the *behavioral intention* construct suggests that the constructs included were able to capture substantial aspects of acceptance.

## 8 Conclusion

This study contributes to the understanding of acceptance factors in the field of telerehabilitation. It highlights that *performance expectancy*, *hedonic motivation* and *habit* are key predictors of *behavioral intention* and, thus, attitudinal acceptance. These aspects can be specifically targeted and supported in rehabilitation centers in order to promote acceptance among rehabilitation patients.

The UTAUT2 model, which is often applied in the context of e-health and telerehabilitation without modification, should not be adopted uncritically. Our findings suggest that not all dimensions of the model necessarily make a significant contribution to the acceptance of telerehabilitation. Therefore, further research is needed to confirm which of the UTAUT constructs influence the acceptance of telerehabilitation. Particular attention should be paid to the determinants of *use behavior*.

In addition, a valid instrument for measuring *use behavior* needs to be developed. This should also take into account the specific circumstances of the rehabilitation context, such as the externally regulated nature of telerehabilitation, for example therapy plans or prescription-related frequency restrictions.

## Data Availability

The original contributions presented in the study are included in the article/Supplementary Material, further inquiries can be directed to the corresponding author.
