# Supplementary material for: User acceptance of telerehabilitation in Germany: a structural equation modeling approach based on the UTAUT2 model
Source: Front Digit Health. 2026 Jul 1;8:1699317. doi: 10.3389/fdgth.2026.1699317 (PMC13386417; doi:10.3389/fdgth.2026.1699317)
Supplement: Supplementary file 1 [file Datasheet2.docx]

**Additional File 2**

**Table A2.1: coefficients - bias-corrected confidence intervals**

|  | Original data set (O) | Sample mean value (M) | Bias | 2.5% | 97.5% |
| --- | --- | --- | --- | --- | --- |
| Effort Expectancy -> Behavioral Intention | -0.012 | -0.010 | 0.001 | -0.149 | 0.121 |
| Facilitating Conditions -> Behavioral Intention | 0.007 | 0.011 | 0.004 | -0.089 | 0.100 |
| Facilitating Conditions -> Use Behavior | 0.029 | 0.023 | -0.006 | -0.157 | 0.222 |
| Habit -> Behavioral Intention | 0.340 | 0.339 | -0.000 | 0.207 | 0.478 |
| Habit -> Use Behavior | -0.286 | -0.286 | -0.000 | -0.541 | -0.025 |
| Hedonic Motivation -> Behavioral Intention | 0.300 | 0.296 | -0.004 | 0.151 | 0.479 |
| Behavioral Intention ->  Use Behavior | 0.074 | 0.077 | 0.003 | -0.178 | 0.319 |
| Performance Expectancy -> Behavioral Intention | 0.298 | 0.296 | -0.001 | 0.174 | 0.429 |
| Privacy Concern -> Behavioral Intention | 0.016 | 0.014 | -0.001 | -0.052 | 0.089 |
| Social Influence -> Behavioral Intention | 0.003 | 0.006 | 0.003 | -0.083 | 0.094 |

**Table A2.2: HTMT - bias-corrected confidence intervals**

|  | Original data set (O) | Sample mean value (M) | Bias | 5.0% | 95.0% |
| --- | --- | --- | --- | --- | --- |
| Facilitating Conditions <-> Effort Expectancy | 0.781 | 0.783 | 0.002 | 0.680 | 0.869 |
| Habit <->  Effort Expectancy | 0.539 | 0.537 | -0.002 | 0.420 | 0.640 |
| Habit <->  Facilitating Conditions | 0.466 | 0.466 | 0.000 | 0.350 | 0.572 |
| Hedonic Motivation <-> Effort Expectancy | 0.654 | 0.653 | -0.002 | 0.548 | 0.743 |
| Hedonic Motivation <-> Facilitating Conditions | 0.554 | 0.555 | 0.001 | 0.435 | 0.659 |
| Hedonic Motivation <-> Habit | 0.881 | 0.880 | -0.000 | 0.828 | 0.918 |
| Behavioral Intention <-> Effort Expectancy | 0.525 | 0.525 | -0.001 | 0.388 | 0.647 |
| Behavioral Intention <-> Facilitating Conditions | 0.444 | 0.446 | 0.001 | 0.323 | 0.552 |
| Behavioral Intention <-> Habit | 0.878 | 0.878 | 0.000 | 0.832 | 0.915 |
| Behavioral Intention <-> Hedonic Motivation | 0.849 | 0.848 | -0.001 | 0.794 | 0.891 |
| Performance Expectancy <->  Effort Expectancy | 0.532 | 0.530 | -0.002 | 0.407 | 0.644 |
| Performance Expectancy <-> Facilitating Conditions | 0.423 | 0.424 | 0.001 | 0.295 | 0.547 |
| Performance Expectancy <-> Habit | 0.818 | 0.817 | -0.001 | 0.753 | 0.869 |
| Performance Expectancy <-> Hedonic Motivation | 0.803 | 0.801 | -0.002 | 0.728 | 0.859 |
| Performance Expectancy <-> Behavioral Intention | 0.834 | 0.832 | -0.001 | 0.766 | 0.883 |
| Privacy Concern <->  Effort Expectancy | 0.327 | 0.327 | -0.000 | 0.206 | 0.439 |
| Privacy Concern <-> Facilitating Conditions | 0.347 | 0.347 | 0.000 | 0.215 | 0.467 |
| Privacy Concern <-> Habit | 0.176 | 0.178 | 0.002 | 0.062 | 0.300 |
| Privacy Concern <->  Hedonic Motivation | 0.218 | 0.219 | 0.001 | 0.103 | 0.332 |
| Privacy Concern <-> Behavioral Intention | 0.149 | 0.153 | 0.004 | 0.051 | 0.270 |
| Privacy Concern <-> Performance Expectancy | 0.143 | 0.148 | 0.005 | 0.051 | 0.266 |
| Social Influence <->  Effort Expectancy | 0.372 | 0.375 | 0.003 | 0.249 | 0.497 |
| Social Influence <-> Facilitating Conditions | 0.442 | 0.443 | 0.001 | 0.304 | 0.564 |
| Social Influence <-> Habit | 0.740 | 0.742 | 0.002 | 0.632 | 0.824 |
| Social Influence <->  Hedonic Motivation | 0.654 | 0.655 | 0.000 | 0.549 | 0.743 |
| Social Influence <-> Behavioral Intention | 0.653 | 0.655 | 0.002 | 0.544 | 0.739 |
| Social Influence <-> Performance Expectancy | 0.683 | 0.683 | 0.000 | 0.586 | 0.763 |
| Social Influence <->  Privacy Concern | 0.130 | 0.145 | 0.014 | 0.052 | 0.250 |
| Use Behavior <-> Effort Expectancy | 0.156 | 0.163 | 0.007 | 0.056 | 0.285 |
| Use Behavior <->  Facilitating Conditions | 0.060 | 0.109 | 0.049 | 0.008 | 0.112 |
| Use Behavior <-> Habit | 0.225 | 0.225 | -0.000 | 0.087 | 0.351 |
| Use Behavior <-> Hedonic Motivation | 0.171 | 0.172 | 0.001 | 0.046 | 0.310 |
| Use Behavior <->  Behavioral Intention | 0.154 | 0.159 | 0.005 | 0.060 | 0.276 |
| Use Behavior <-> Performance Expectancy | 0.110 | 0.120 | 0.010 | 0.027 | 0.243 |
| Use Behavior <->  Privacy Concern | 0.020 | 0.060 | 0.041 | 0.002 | 0.030 |
| Use Behavior <->  Social Influence | 0.073 | 0.101 | 0.028 | 0.014 | 0.161 |
