# Supplementary material for: User acceptance of telerehabilitation in Germany: a structural equation modeling approach based on the UTAUT2 model
Source: Front Digit Health. 2026 Jul 1;8:1699317. doi: 10.3389/fdgth.2026.1699317 (PMC13386417; doi:10.3389/fdgth.2026.1699317)
Supplement: Supplementary file 2 [file Datasheet1.docx]

**Additional File 1**

**Table A1.1: Type of telerehabilitation**

| Type | N | % |
| --- | --- | --- |
| App | 185 | 80.4% |
| Internet portal/web platform of the rehabilitation facility | 35 | 15.2% |
| Video conferencing program | 2 | 0.9% |
| Others | 8 | 3.5% |
| Total | 230 | 100% |

**Table A1.2: Purposes of use; multiple answers were possible (n=230)**

| Purpose of use | N | % of cases |
| --- | --- | --- |
| Exercising | 186 | 80.9% |
| Video consultations | 8 | 3.5% |
| Exchange in a chat (with therapists or other rehabilitation patients) | 81 | 35.2% |
| Training courses | 134 | 58.3% |
| Information gathering | 106 | 46.1% |
| Behavioral change | 42 | 18.3% |
| Self-management | 30 | 13.0% |
| Planning therapy sessions | 17 | 7.4% |
| Other purposes | 18 | 7.8% |
| Total | 622 | 270.4% |

**Table A1.3: Population size**

| Population | N | % |
| --- | --- | --- |
| < 5.000 inhabitants | 55 | 23.9% |
| 5.001 bis 50.000 | 96 | 41.7% |
| 50.001 bis 100.000 | 24 | 10.4% |
| > 100.000 | 41 | 17.8% |
| I prefer not to answer. | 11 | 4.8% |
| No response | 3 | 1.3% |
| Total | 230 | 100.0% |

**Table A1.4: Use of telerehabilitation services at the present time**

| Use at present | N | % |
| --- | --- | --- |
| Yes | 212 | 92,2% |
| No | 18 | 7,8% |
| Total | 230 | 100% |

**Table A1.5: Duration of telerehabilitation use during a session**

| Duration of use | N | % |
| --- | --- | --- |
| < 10 minutes | 6 | 2,6% |
| 10 to 30 minutes | 82 | 35,7% |
| 31 to 60 minutes | 120 | 52,2% |
| 61 to 90 minutes | 20 | 8,7% |
| > 90 minutes | 2 | 0,9% |
| Total | 230 | 100% |

**Table A1.6: Frequency of use (use behavior)**

| Frequency | N | % |
| --- | --- | --- |
| I only tested it once. | 4 | 1,7% |
| Once a month | 1 | 0,4% |
| Several times a month | 20 | 8,7% |
| Once a week | 41 | 17,8% |
| Several times a week | 138 | 60,0% |
| Once a day | 20 | 8,7% |
| Several times a day | 6 | 2,6% |
| Total | 230 | 100% |
