# Supplementary material for: User acceptance of telerehabilitation in Germany: a structural equation modeling approach based on the UTAUT2 model
Source: Front Digit Health. 2026 Jul 1;8:1699317. doi: 10.3389/fdgth.2026.1699317 (PMC13386417; doi:10.3389/fdgth.2026.1699317)
Supplement: Supplementary file 3 [file Datasheet3.docx]

**Additional File 3**

**Table A3.1: Multigroup Analysis – MICOM Step 2**

|  | Original correlations | Correlation mean value of the permutations | 5.0% | Permutation *p*-values |
| --- | --- | --- | --- | --- |
| Effort Expectancy | 0.999 | 0.997 | 0.990 | 0.658 |
| Facilitating Conditions | 0.994 | 0.982 | 0.935 | 0.613 |
| Habit | 1.000 | 1.000 | 0.999 | 0.880 |
| Hedonic Motivation | 1.000 | 1.000 | 1.000 | 0.467 |
| Behavioral Intention | 0.999 | 1.000 | 1.000 | 0.044 |
| Performance Expectancy | 1.000 | 1.000 | 1.000 | 0.857 |
| Privacy Concern | 0.998 | 0.980 | 0.931 | 0.448 |
| Social Influence | 1.000 | 0.995 | 0.981 | 0.995 |
| Use Behavior | 1.000 | 1.000 | 1.000 | 0.106 |
